# Surfactant replacement therapy in combination with different non-invasive ventilation techniques in spontaneously-breathing, surfactant-depleted adult rabbits

**DOI:** 10.1371/journal.pone.0200542

**Published:** 2018-07-12

**Authors:** Francesca Ricci, Costanza Casiraghi, Matteo Storti, Francesco D’Alò, Chiara Catozzi, Roberta Ciccimarra, Francesca Ravanetti, Antonio Cacchioli, Gino Villetti, Maurizio Civelli, Xabi Murgia, Virgilio Carnielli, Fabrizio Salomone

**Affiliations:** 1 Chiesi Farmaceutici, R&D Department, Parma, Italy; 2 Department of Veterinary Science, University of Parma, Parma, Italy; 3 Department of Drug Delivery, Helmholtz Institute for Pharmaceutical Research Saarland, Saarbrücken, Germany; 4 Division of Neonatology, Polytechnic University of Marche and Salesi Children’s Hospital, Ancona, Italy; University of Bari, ITALY

## Abstract

Nasal intermittent positive pressure ventilation (NIPPV) holds great potential as a primary ventilation support method for Respiratory Distress Syndrome (RDS). The use of NIPPV may also be of great value combined with minimally invasive surfactant delivery. Our aim was to implement an *in vivo* model of RDS, which can be managed with different non-invasive ventilation (NIV) strategies, including non-synchronized NIPPV, synchronized NIPPV (SNIPPV), and nasal continuous positive airway pressure (NCPAP). Forty-two surfactant-depleted adult rabbits were allocated in six different groups: three groups of animals were treated with only NIV for three hours (NIPPV, SNIPPV, and NCPAP groups), while three other groups were treated with surfactant (SF) followed by NIV (NIPPV+SF, SNIPPV+SF, and NCPAP+SF groups). Arterial gas exchange, ventilation indices, and dynamic compliance were assessed. Post-mortem the lungs were sampled for histological evaluation. Surfactant depletion was successfully achieved by repeated broncho-alveolar lavages (BALs). After BALs, all animals developed a moderate respiratory distress, which could not be reverted by merely applying NIV. Conversely, surfactant administration followed by NIV induced a rapid improvement of arterial oxygenation in all surfactant-treated groups. Breath synchronization was associated with a significantly better response in terms of gas exchange and dynamic compliance compared to non-synchronized NIPPV, showing also the lowest injury scores after histological assessment. The proposed *in vivo* model of surfactant deficiency was successfully managed with NCPAP, NIPPV, or SNIPPV; this model resembles a moderate respiratory distress and it is suitable for the preclinical testing of less invasive surfactant administration techniques.

## Introduction

Preterm birth is characterized by a marked immaturity of all organ systems. In very preterm infants, the signs of lung immaturity manifest shortly after birth. Alveolar instability, arising from the incapacity of the underdeveloped alveolar epithelium to synthesize and secrete adequate quantity of surfactant [1,2], leads to alveolar collapse, which in turn compromises the vital systemic oxygen delivery and, ultimately, the life of infants who suffer the so called respiratory distress syndrome (RDS).

RDS infants routinely require artificial respiratory support and surfactant replacement therapy [3,4]. The classic treatment against RDS entailed early tracheal intubation of the infants followed by the intratracheal instillation of exogenous surfactant and a variable period of intensive mechanical ventilation [5,6]. Although this approach has proved to be a life-saving therapy, the association between mechanical ventilation through an endotracheal tube and the incidence of chronic lung disease [7–9] led to the search of alternative, non-invasive ventilation (NIV) strategies.

Nasal continuous positive airway pressure (NCPAP) has been the earliest form of non-invasive respiratory support [10,11]. Its use as a first line treatment against RDS (without surfactant treatment) has become nowadays standard clinical practice [3,12]. However, despite its efficacy, this technique cannot always avoid tracheal intubation, particularly for very low-gestational-age preterm infants [13,14]. Nasal intermittent positive pressure ventilation (NIPPV) is an enhanced form of NCPAP, which superimposes a pre-set number of mandatory “breaths” to the background NCPAP [15]. Moreover, it is possible to synchronize the mandatory “breaths” provided by the ventilator with the spontaneous breathing efforts of the infant, as in synchronized NIPPV (SNIPPV) [16]. Breath synchronization further improves pulmonary mechanics, reduces the work of breathing, and enhances gas exchange [17].

A limitation regarding the universal application of NIV to all preterm infants is that newborns with moderate to severe RDS will be inadequately supported by NIV, ultimately requiring intubation and surfactant therapy at a later stage [18]. The solution to avoid the failure of NIV is the timely administration of exogenous surfactant. Recently, successful approaches of less invasive surfactant administration have been described, consisting of delivering an intratracheal bolus of surfactant through a thin tube, while infants are managed with NCPAP [19,20]. Surfactant aerosol delivery in combination with NCPAP has been investigated as the ideal, non-invasive alternative which holds the potential to couple NIV with surfactant replacement [21–26]. Nevertheless, the technical challenge of delivering medical aerosols to neonates is high [27] and needs further preclinical development, including appropriate *in vivo* experiments with models that can be managed with NIV.

In a previous study, we have implemented and fully characterized the spontaneously-breathing, lung-lavaged adult rabbit as an *in vivo* model of respiratory distress which can be managed with NCPAP [28]. Interestingly, this model showed a significant improvement of the pulmonary status if surfactant therapy was applied in combination with NCPAP compared to NCPAP only. In the present study, our primary aim was to address the feasibility of implementing this model with alternative techniques of NIV such as NIPPV and SNIPPV. In addition, we hypothesized that the use of NIPPV or SNIPPV could improve the pulmonary outcomes after surfactant treatment compared to NCPAP.

## Materials and methods

### Animal handling and surfactant delivery protocol

The experiments were carried out in 6- to 7-week-old rabbits. The experimental procedure was approved by the intramural Animal Welfare Body and the Italian Ministry of Health (Prot.n° 1300-2015-PR) and complied with the European and Italian regulations for animal care.

Rabbits (body weight of 1.5–2.5 kg) were initially sedated with intramuscular (i.m.) medetomidine (Domitor^®^, 2 mg/kg) and handled as previously described [28]. Briefly, the throat of the animals was first shaved and local anaesthesia was applied in the anterior neck with lidocaine gel (Luan^®^ 2.5%). Thirty minutes later the animals received 50 mg/kg of ketamine (Imalgene 1000^®^, Merial-Boehringer Ingelheim, France) and 5 mg/kg of xylazine (Rompun^®^, Bayer, Germany) i.m. Rabbits, in supine position, were intubated and stabilized on positive pressure ventilation (Fabian HFO, Acutronic, Zug, Switzerland) with the following settings: FiO_2_ = 100%, Flow = 10 L/min, respiratory rate (RR) = 40 breaths/min, positive end-expiratory pressure (PEEP) = 3 cmH_2_O, tidal volume (V_T_) targeted to 7 ml/kg (with the peak inspiratory pressure, PIP, not exceeding 15 cmH_2_O) and inspiratory time of 0.5 sec. Airway flow, mean airway pressure (MAP) and V_T_ were monitored with a flow sensor connected to the endotracheal tube, as long as the animals were intubated. Body temperature was monitored with a rectal probe and it was maintained by placing a heating pad underneath the animal.

After endotracheal intubation, a catheter was inserted into the right jugular vein for continuous infusion of 1 mg/ml of ketamine and 0.1 mg/ml of xylazine (100 μl/min). Trometamol (tris-hydroxymethyl aminomethane, THAM, 1M, Sigma-Aldrich, USA) was also infused during the surfactant depletion procedure. A second catheter was inserted into the right carotid artery for blood sampling. After instrumentation, the baseline blood gases were measured with an emogas analyzer (Radiometer Medical, Denmark).

If the initial inclusion criteria of arterial oxygen partial pressure (PaO_2_) value > 400 mmHg at PIP < 15 cmH_2_O were met, the animal was included in the study. Repeated broncho-alveolar lavages (BALs) were performed by flushing the airways with 20 ml/kg of pre-warmed (37°C) 0.9% NaCl solution, followed by a short recovery period in-between, until a PaO_2_ value < 100 mmHg was reached. Then, if after 15 min of stabilization on mechanical ventilation the respiratory failure was confirmed (PaO_2_ < 100 mmHg, with PIP not exceeding 23 cmH_2_O), the animal was allocated in one of the study groups. NIV support was applied using nasal prongs (Fisher & Paykel, Auckland, New Zealand). Just before extubation, the animals assigned to the surfactant + NIV groups received a bolus of 200 mg/kg of surfactant (SF, Curosurf, Chiesi Farmaceutici, Parma, Italy).

Animals included in the **NCPAP** (n = 6) and **NCPAP+SF** (n = 6) groups were maintained in NCPAP (5 cmH_2_O, Fabian HFO, Acutronic, Zug, Switzerland) for 180 minutes, as previously described [28].

The animals included in the **NIPPV** (n = 8) and **NIPPV+SF** (n = 7) groups were managed with non-synchronized NIPPV (Fabian HFO, Acutronic, Zug, Switzerland) for 180 min. The initial settings of NIPPV support were: PIP = 21–23 cmH_2_O, PEEP 5 cmH_2_O, RR (guaranteed) = 60 breaths/min, inspiratory time = 0.5.

The **SNIPPV** (n = 7) and **SNIPPV+SF** (n = 8) groups were managed with SNIPPV using the Sophie ventilator (Fritz Stephan GmbH, Gackenbach, Germany) with the Graseby abdominal capsule-triggering device (Fritz Stephan GmbH, Gackenbach, Germany) for breath synchronization. Briefly, this system detects the diaphragmatic mechanical perturbations induced by the animals’ respiratory effort, which are then converted into a stable, fast-reacting (<30 ms) trigger signal. The initial settings of SNIPPV support were: PIP = 20 cmH_2_O, PEEP 5 cmH_2_O, RR (guaranteed) = 60 breaths/min, inspiratory time = 0.5.

### Gas exchange

Arterial carbon dioxide partial pressure (PaCO_2_) and PaO_2_ were measured right after the induction of the anaesthesia (baseline), after inducing the respiratory failure by repeated BALs, and after the stabilization period following the insult to confirm the respiratory failure (Radiometer Medical, Denmark). Arterial blood gases were also measured right after placing the animals on NIV support, 15 and 30 min after the start of NIV support, and then every 30 min until the end of the experiment.

### Ventilation indices

The ventilation efficacy index (VEI) and the Oxygenation index (OI) were calculated at baseline, after surfactant depletion by repeated BALs, and at the end of the observational period. Once the observational period was completed, animals were shifted from NIV to invasive mechanical ventilation for a brief period of time, with the same settings used at baseline (before the BALs: FiO_2_ 100%, Flow = 10 L/min; RR = 40 bpm, PEEP = 3 cmH_2_O, V_T_ targeted to 7 ml/kg, and inspiratory time of 0.5).

VEI was calculated to evaluate the overall ventilation efficiency of mechanically ventilated animals independently from the ventilation settings, as follows:

$$VEI=\frac{3800}{\left(PIP÷PEEP)\times RR\times Pa(carbon\;dioxide\right)}$$

OI was calculated to quantify the severity of pulmonary dysfunction in ventilated animals:

$$OI=\frac{\left[Fi(oxygen)\times MAP\times 100\right]}{Pa(oxygen)}$$

### Lung mechanics

Dynamic compliance (C_dyn_) was determined in all animals at baseline, after BAL-induced surfactant depletion, and again at the end of the follow-up period, after re-intubation. C_dyn_ was calculated by dividing the changes in lung volume (ΔV) by the changes in pressure (ΔP) multiplied by the weight of the animal in Kg. ΔV and ΔP were obtained by spirometry (Acutronic).

$$Cdyn=\frac{\Delta V}{\Delta P\times weight}$$

### Histological analysis

The right lung was sampled in the cranial, middle and caudal lobes to represent non-dependent, intermediate and dependent lung regions. The samples were fixed in 10% neutral buffered formalin, then dehydrated in graded alcohol solutions, xylene clarified, paraffin infiltrated by means of an automatic processor (ATP 700 Tissue Processor, Histo-line laboratories, Italy), and embedded with the dorsal surface of the slice down (EG 1160, Leica Biosystems, Mannheim, Germany). Sections, 5 μm thick, were obtained using a rotary microtome (Slee Cut 6062, Slee Medical, Mainz, Germany). Three slides for each lung region were then further deparaffinized, rehydrated in descending grades of ethanol, and finally stained with hematoxylin and eosin (Sigma). Images of the samples were acquired at 40X magnification by a digital slide scanner (NanoZoomer, Hamamatsu, Hamamatsu City, Japan). Lung injury was scored by an investigator blinded to the experimental design using a semi-quantitative scoring system [29,30]. All parenchyma within a histological slide was scored considering multiple and not overlapping ROIs at 10X magnification. According to internal standard procedure, the ROIs occupied for more than 30% in area by conductive airways or blood vessels were excluded. Alveolar and interstitial inflammation, alveolar and interstitial hemorrhage, edema, and atelectasis were each scored on a 0 to 4 point scale. Each ROI was scored as 0 if the item was absent or normal, as 1 if the item was present in 25% of the field, as 2 if it was present in 50% of the field, as 3 if it was present in 75% of the field and as 4 if the item was apparent throughout the whole field. The total injury score was calculated as a sum of these scores and the average of the ROIs was reported for each section.

### Statistical analysis

Physiological parameters are presented as mean ± SEM. Raw data were analyzed and compared by repeated measures two-way analysis of variance (ANOVA) as a function of group and time or one-way ANOVA, followed by Tukey’s post-hoc test. The data of the histological score are presented as mean ± SEM and analysed by the non-parametric Kruskal-Wallis test. Statistical analysis was performed using GraphPad software, version 7.0.

## Results

The body weight of the animals, the PaO_2_ and the C_dyn_ at baseline and the number of BALs needed to achieve respiratory failure were not significantly different between the groups (Table 1). BALs led to a marked (*P*<0.0001) decrease of PaO_2_ and C_dyn_ values, indicative of a dramatic reduction of the alveolar surfactant pool [28].

**Table 1:** Body weight, number of BALs, and baseline as well as post-BALs (respiratory failure) values of PaO_2_ and C_dyn_ in adult rabbits included in the study.

Group	Body weight (Kg)	Number of BALs	Baseline PaO_2_ (mmHg)	Post-BALs PaO_2_ (mmHg)	Baseline Cdyn (ml/cmH_2_O/kg)	Post-BALs Cdyn (ml/cmH_2_O/kg)
**NCPAP**	1.85 ± 0.06	9.83 ± 1.13	445 ± 20	72 ± 23*	0.94 ± 0.05	0.33 ± 0.05*
**NCPAP+SF**	1.75 ± 0.08	9.33 ± 1.08	420 ± 12	58 ± 8*	0.88 ± 0.06	0.31 ± 0.06*
**NIPPV**	1.90 ± 0.06	9.00 ± 0.89	446 ± 15	65 ± 5*	1.11 ± 0.37	0.37 ± 0.07*
**NIPPV+SF**	1.91 ± 0.05	10.1 ± 1.30	437 ± 8.84	56 ± 4*	1.12 ± 0.10	0.29 ± 0.03*
**SNIPPV**	1.86 ± 0.06	8.83 ± 0.94	440 ± 10	48 ± 7*	0.82 ± 0.02	0.42 ± 0.07*
**SNIPPV+SF**	1.70 ± 0.06	8.85 ± 1.33	434 ± 13	61 ± 15*	0.90 ± 0.09	0.32 ± 0.04*

Nasal continuous positive airway pressure, NCPAP; surfactant, SF; nasal intermittent positive pressure ventilation, NIPPV; synchronized NIPPV, SNIPPV; arterial oxygen partial pressure, PaO_2_; dynamic compliance, C_dyn_.

* *P* vs. baseline values < 0.0001.

### Gas exchange

**Fig 1:**
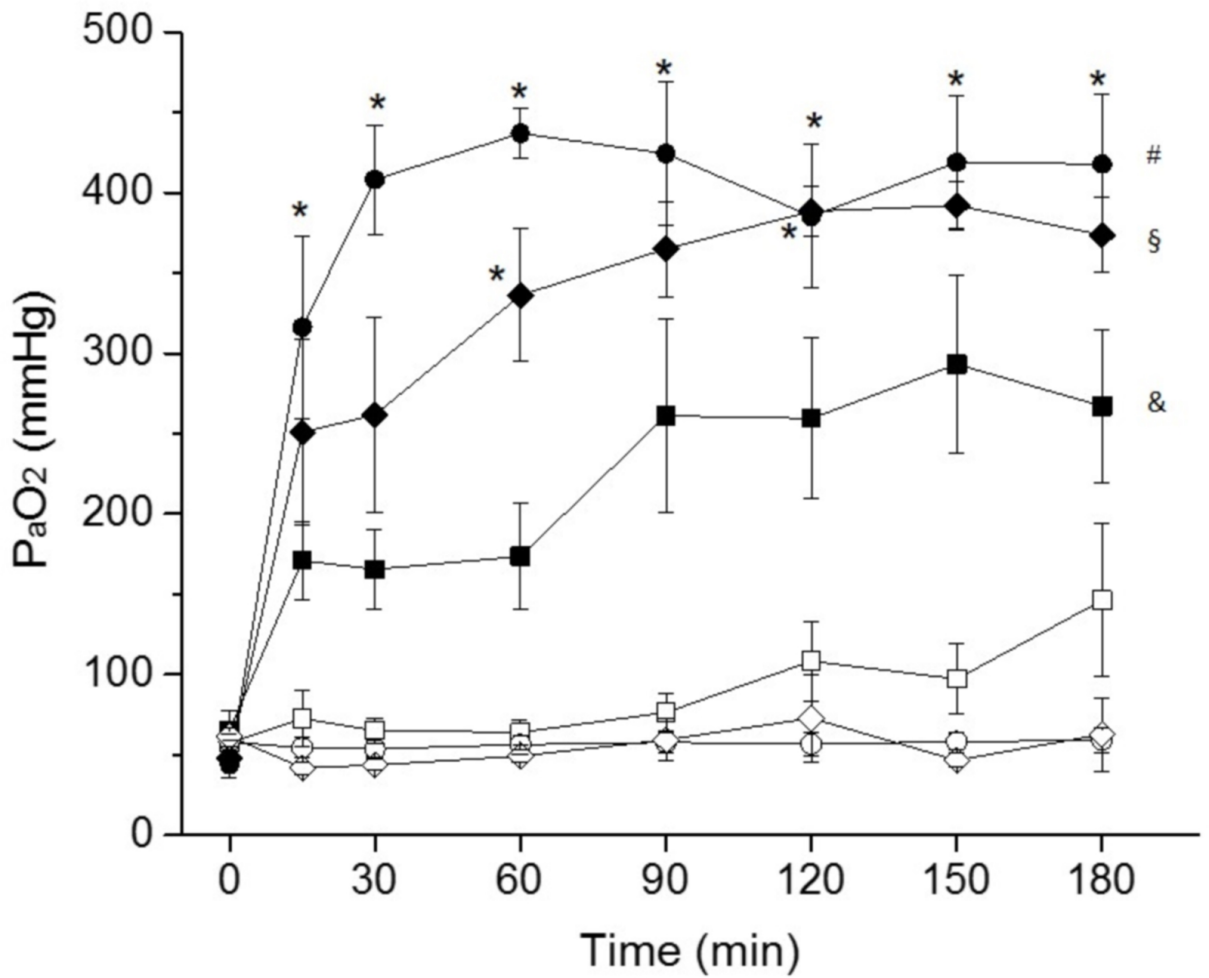
Mean PaO_2_ values over time of surfactant-depleted rabbits managed with non-invasive ventilation. Surfactant-depleted adult rabbits were treated with nasal continuous positive pressure ventilation (NCPAP, white diamonds), with NCPAP + intratracheal surfactant (NCPAP+SF, black diamonds), with nasal non-synchronized intermittent positive ventilation (NIPPV, white squares), with NIPPV + intratracheal surfactant (NIPPV+SF, black squares), with Synchronized Intermittent Positive Pressure Ventilation (SNIPPV, white circles), or with SNIPPV + intratracheal surfactant (SNIPPV+SF, black circles). Surfactant-treated animals (black symbols) received a bolus of surfactant before extubation, immediately after the 0 time-point. Values are shown as the mean ± SEM. * *P* vs. NIPPV+SF group < 0.01; ^#^ *P* vs. SNIPPV group < 0.01; ^§^ *P* vs. NCPAP group < 0.01; ^&^ *P* vs. NIPPV group < 0.01.

After surfactant depletion, all groups showed mean PaO_2_ values below 80 mmHg, even though the FiO_2_ was set to 100%. As expected, PaO_2_ values increased in all SF-treated groups immediately after SF instillation (Fig 1). In particular, PaO_2_ values were back to the basal levels (before BALs) after 60 minutes in the SNIPPV+SF group and after 120 minutes in the NCPAP+SF group. Arterial oxygenation was clearly superior in SF-treated animals throughout the follow up period. Among SF-treated groups, the highest mean PaO_2_ values were observed for the SNIPPV+SF group, which were significantly higher compared to NIPPV+SF at any time-point. The mean PaO_2_ values of the NCPAP+SF group were higher than the values observed for NIPPV, reaching statistical significance at 60 and 120 minutes.

**Fig 2:**
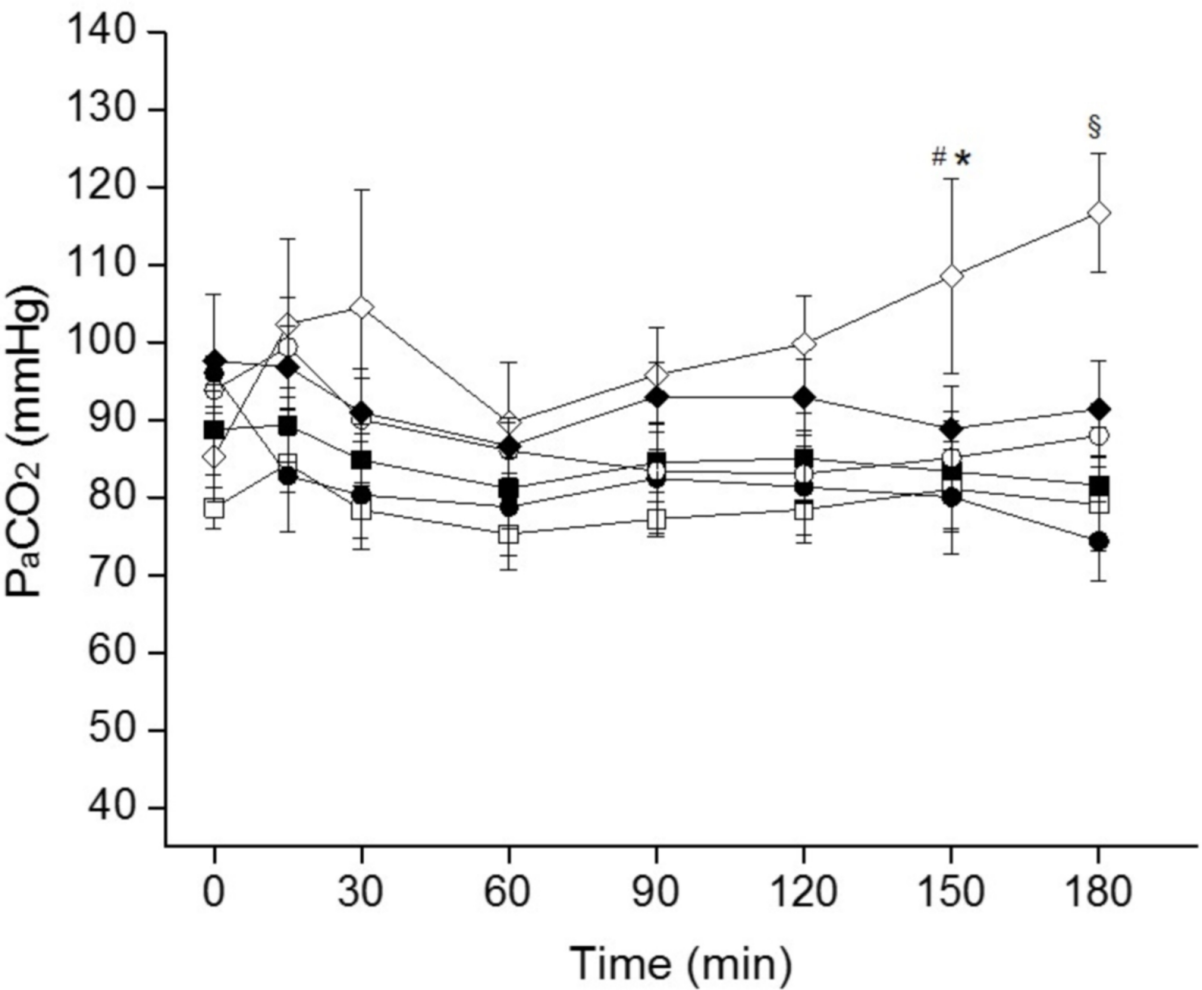
Mean PaCO_2_ values over time of surfactant-depleted rabbits managed with non-invasive ventilation. Surfactant-depleted adult rabbits were treated with NCPAP only (white diamonds), with NCPAP+SF (black diamonds), with NIPPV only (white squares), with NIPPV+SF (black squares), with SNIPPV only (white circles), or with SNIPPV+SF (black circles). Surfactant-treated animals (black symbols) received a bolus of surfactant before extubation, immediately after the 0 time-point. Values are shown as the mean ± SEM * *P* vs. NIPPV group < 0.01; ^#^ *P* vs. NIPPV+SF group < 0.01; ^§^ *P* vs. all other groups < 0.01.

The mean PaCO_2_ values increased in all groups due to the respiratory failure induced by the BALs. Even though a slight downward trend of the mean PaCO_2_ values could be observed within the first 30 min in the SF-treated groups, the arterial carbon dioxide levels remained rather high in all groups, without significant differences in the first two hours of the follow up period. The worst PaCO_2_ values were observed in the NCPAP group; in the last hour of the follow up the mean PaCO_2_ of the NCPAP group was over 100 mmHg, significantly higher compared to NIPPV and NIPPV+SF groups at 150 minutes and to all the other groups at 180 minutes (Fig 2).

### Ventilation indices

To directly compare the pulmonary status of the animals before and after treatment, at the end of NIV follow-up period, all animals were re-intubated and mechanically ventilated with the same ventilation settings as at baseline (before the BALs). The OI and the VEI were therefore calculated for the time-points in which the animals were managed with invasive mechanical ventilation: 1) at baseline, 2) after surfactant depletions by repeated BALs, and 3) at the end of the experiment (180 minutes).

**Fig 3:**
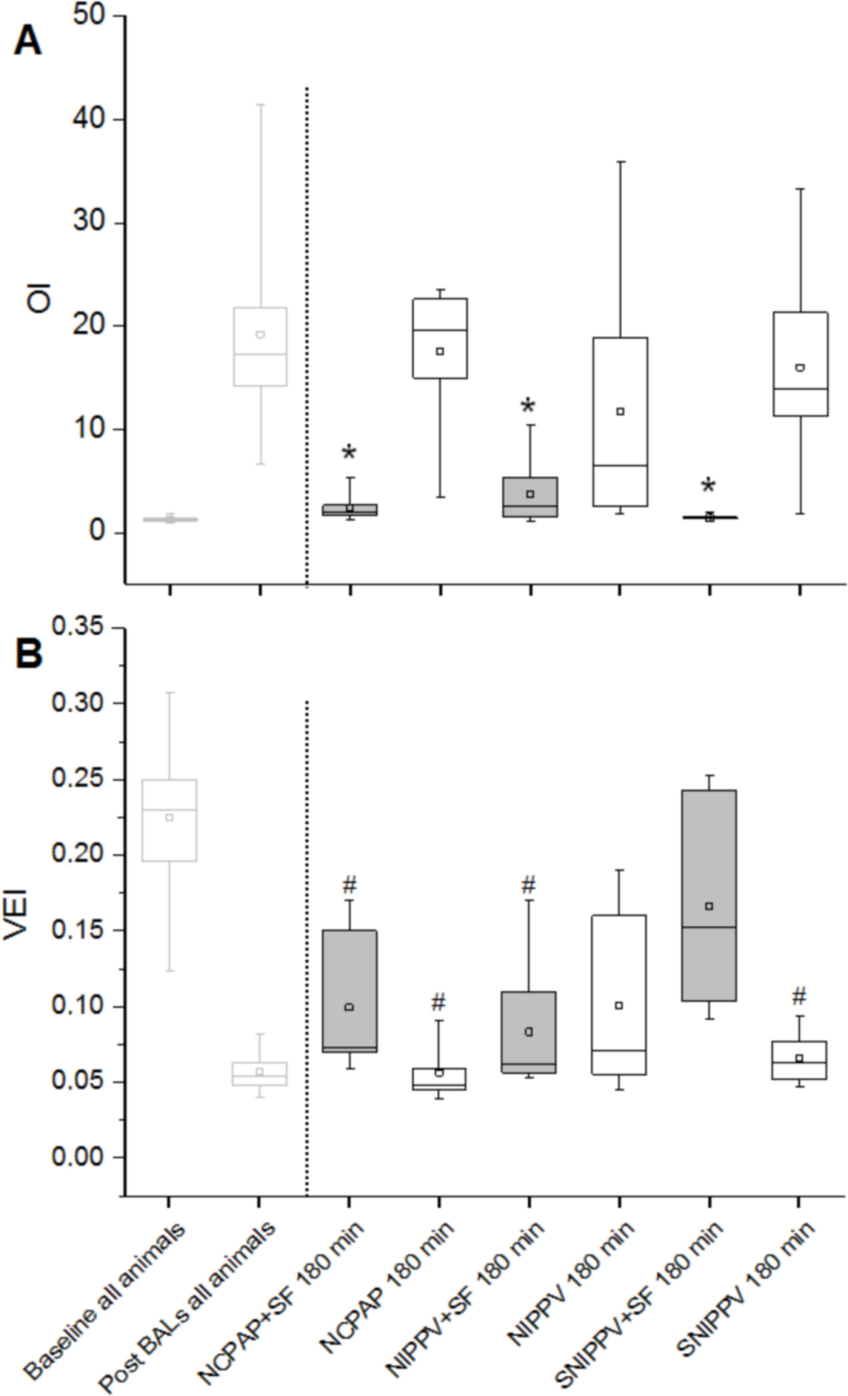
The oxygenation index (OI) and the ventilation efficacy index (VEI) of surfactant-depleted rabbits managed with non-invasive ventilation. To the left of the dashed line, the baseline and post-surfactant depletion OI (A) and VEI (B) values of all the animals featured in the study are given. To the right of the dashed line, the OI and VEI values of the different groups after 180 minutes of management with NIV are shown. Surfactant-treated groups are represented by the box-plots with a grey filling, whereas the groups treated with NIV only are represented by the white box-plots. The small squares within each box-plot indicate the mean of the group. The whiskers indicate the maximum and minimum values observed for each group. * *P* < 0.01 vs. any group not treated with surfactant; ^#^ *P* < 0.01 vs. SNIPPV+SF group. NCPAP: nasal continuous positive airway pressure, SF: surfactant, NIPPV: nasal (non-synchronized) intermittent positive pressure ventilation, and SNIPPV: synchronized NIPPV.

The mean OI at baseline was below 1.4 in all groups, indicative of an optimal pulmonary oxygen exchange. However, surfactant depletion by repeated BALs induced a significant respiratory failure which increased the OI in all animals, yielding a mean value of 19.2 ± 7.6, considering all the animals featured in the study. The tested NIV techniques without SF treatment could not restore the OI to baseline values; moreover, the pulmonary response to the different NIV modalities without surfactant treatment was not uniform within the groups showing a great intra-group variability (Fig 3A). At the end of the experimental period, the mean OI values of the NIPPV, SNIPPV and NCPAP groups were 12.98 ± 4.27 (range 2.46–35.9), 15.17 ± 3.7 (1.76–33.22), and 16.61 ± 2.88 (3.37–22.59), respectively. Surfactant therapy in combination with NIV, however, was associated with a significantly better OI score compared with NIV alone. The combined use of surfactant and SNIPPV or NCPAP could revert the high mean OI values observed after the BALs to baseline values; in addition, it is noteworthy that the pulmonary response to the combined treatment in these two groups was remarkably homogeneous: the mean OI values for the SNIPPV+SF and NCPAP+SF groups were 1.35 ± 0.04 (range 1.15–1.46) and 1.91 ± 0.22 (1.24–2.84), respectively. Surfactant therapy followed by non-synchronized NIPPV also improved the OI significantly compared to animal groups treated with NIV only. Nevertheless, the mean OI of the NIPPV+SF group was slightly higher (4.29 ± 1.18, range 1.57–10.42) than in the SNIPPV+SF and NCPAP+SF groups.

The VEI was markedly influenced by the elevated PaCO_2_ values observed in all groups (Fig 3B). The mean VEI for all the animals at baseline, before the induction of the respiratory failure, was 0.25 ± 0.001, indicative of normal ventilation. The VEI dropped dramatically after the BALs to 0.01 ± 0.001 (*P*<0.001), as expected. At the end of the experiment the highest mean VEI values were achieved by the SNIPPV+SF group (0.17 ± 0.03), which were in turn significantly higher than those achieved by the SNIPPV (0.06 ± 0.01), NCPAP (0.06 ± 0.01), NIPPV+SF (0.08 ± 0.02), and NCPAP+SF (0.10 ± 0.02) groups (*P*<0.01).

### Lung mechanics

**Fig 4:**
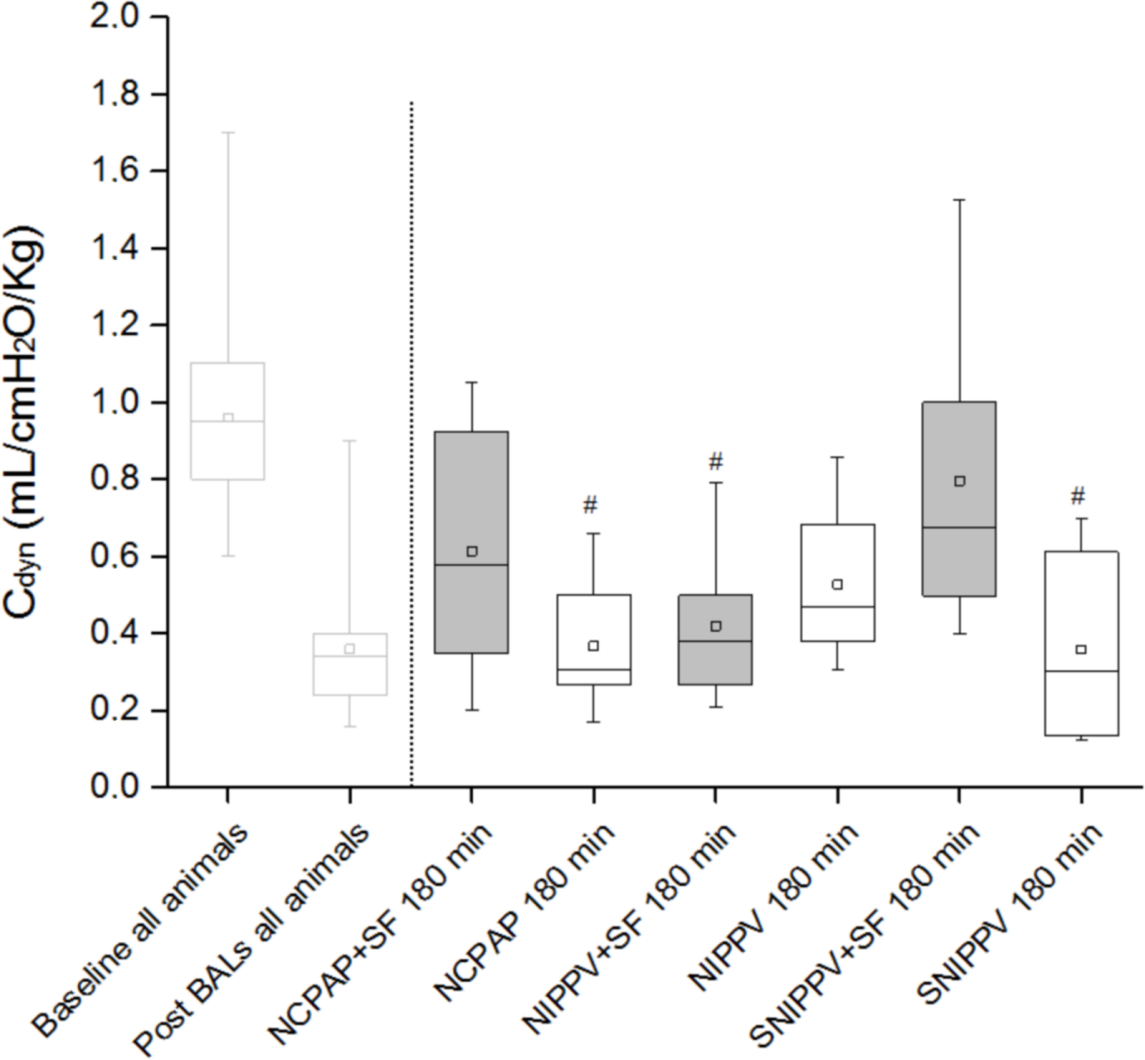
Dynamic compliance (C_dyn_) of surfactant-depleted rabbits managed with non-invasive ventilation. To the left of the dashed line, the baseline and post-surfactant depletion C_dyn_ values of all the animals featured in the study are given. To the right of the dashed line, the C_dyn_ values of the different groups after 180 minutes of management with NIV are shown. Surfactant-treated groups are represented by the box-plots with a grey filling, whereas the groups merely treated with NIV are represented by the white box-plots. The small squares within each box-plot indicate the mean of the group. The whiskers indicate the maximum and minimum values observed for each group. ^#^ *P* < 0.01 vs. SNIPPV+SF group. NCPAP: nasal continuous positive airway pressure, SF: surfactant, NIPPV: nasal (non-synchronized) intermittent positive pressure ventilation, and SNIPPV: synchronized NIPPV.

C_dyn_ values were assessed at those time intervals in which the animals were ventilated with invasive positive pressure ventilation. C_dyn_ was equivalent for all groups at baseline (NIPPV, 1.11 ± 0.1 mL/cmH_2_O/kg; NIPPV+SF, 1.05 ± 0.06 mL/cmH_2_O/kg, SNIPPV, 0.82 ± 0.02 mL/cmH_2_O/kg; SNIPPV+SF, 0.90 ± 0.1 mL/cmH_2_O/kg; NCPAP, 0.94 ± 0.06 mL/cmH_2_O/kg; NCPAP+SF, 0.88 ± 0.07 1.11 ± 0.1 mL/cmH_2_O/kg). However, C_dyn_ dropped dramatically after surfactant depletion to a mean value of 0.36 ± 0.02 mL/cmH_2_O/kg. After three hours of follow-up, mean C_dyn_ values increased in all surfactant-treated groups (Fig 4). The highest mean C_dyn_ value was observed for the SNIPPV+SF group (0.80 ± 0.17 mL/cmH_2_O/kg), which was significantly higher compared to NIPPV+SF, SNIPPV and NCPAP groups (*P*<0.01). In the animal groups treated with NIV only, the mean C_dyn_ values remained low in the SNIPPV and NCPAP groups (0.36 ± 0.08 and 0.37 ± 0.07 mL/cmH_2_O/kg, respectively) and slightly increased in the NIPPV group (0.53 ± 0.07 mL/cmH_2_O/kg).

### Histological analysis

**Fig 5:**
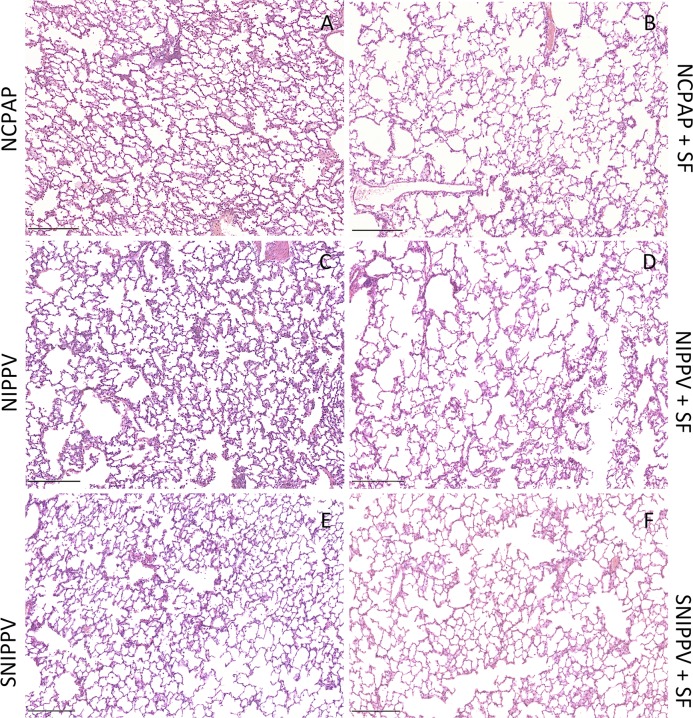
Histological microphotographs of surfactant-depleted rabbits managed with non-invasive ventilation. Histological overview of lung parenchyma presenting representative inflammatory infiltrate, hemorrhage, edema and atelectasis for all groups. SF: surfactant; NCPAP: nasal continuous positive airway pressure (A,B); NIPPV: nasal intermittent positive pressure ventilation (C,D); SNIPPV: synchronized NIPPV (E,F). Haematoxylin & Eosin staining, scale bar 250 μm.

The histological examination of the lungs showed a marked inflammatory interstitial and alveolar neutrophilic infiltration, alveolar and interstitial haemorrhage, thickening of the alveolar wall, and accumulation of proteinaceous oedema and atelectasis: these findings are typical features of an acute inflammation (examples in **S1 Fig**). From the overall view of the samples, surfactant-treated groups showed a moderate decrease of the neutrophil infiltrates and of the haemorrhagic areas both in the alveolar space and interstitium compared to their reference groups treated with NIV only (**Fig 5 and Table 2**).

**Table 2:** Histological scores of surfactant-depleted adult rabbits.

Group	Inflammation	Hemorrhage	Edema	Atelectasis	Sum score
**NCPAP**	3.5 ± 0.2^#^	0.9 ± 0.1^‡^	1.3 ± 0.1	1.5 ± 0.1^#^	7.1 ± 0.5^#^
**NCPAP+SF**	2.8 ± 0.1	0.9 ± 0.1	1.1 ± 0.1*	1.0 ± 0.1	5.6 ± 0.4
**NIPPV**	3.9 ± 0.2^†^	2.3 ± 0.2^†^^§^	1.6 ± 0.1	1.2 ± 0.1	8.9 ± 0.5^‡^^$^
**NIPPV+SF**	3.2 ± 0.2*	1.6 ± 0.2*^#^	1.4 ± 0.1**^#^	1.2 ± 0.1*	7.4 ± 0.6**^#^
**SNIPPV**	3.1 ± 0.2*	1.3 ± 0.2*	1.3 ± 0.1**	1.2 ± 0.1**	6.9 ± 0.5**
**SNIPPV+SF**	2.3 ± 0.2	0.8 ± 0.1	0.8 ± 0.1	0.7 ± 0.1	4.5 ± 0.3

Nasal continuous positive airway pressure, NCPAP; surfactant, SF; nasal intermittent positive pressure ventilation, NIPPV; synchronized NIPPV, SNIPPV.

* *P* vs. SNIPPV+SF < 0.05

** *P* vs. SNIPPV+SF < 0.01

# *P* vs. NCPAP+SF < 0.05

† *P* vs. SNIPPV < 0.05

‡ *P* vs. SNIPPV < 0.01

$ *P* vs. NCPAP < 0.05

§ *P* vs. NCPAP < 0.01.

The sum score for SNIPPV+ SF and NCPAP+SF groups was significantly lower compared to the SNIPPV and NCPAP groups, respectively. Among surfactant-treated groups, SNIPPV+SF and NCPAP+SF performed significantly better than NIPPV+SF. Indeed, the SNIPPV+SF group had the lowest scores among all groups.

Irrespective of whether surfactant was administered or not, synchronization of the animal’s breathing pattern and the ventilator’s drive during nasal intermittent ventilation was associated with significantly better histological outcomes.

## Discussion

In the present study, we have successfully implemented an *in vivo* model of surfactant depletion induced by repeated BALs, which can be managed with different NIV techniques, including NCPAP, NIPPV, and SNIPPV. As evidenced by the marked drop of arterial oxygenation and C_dyn_, the respiratory distress induced to the animals after lung lavage was severe enough to preclude the full pulmonary recovery by merely applying NIV. However, early surfactant instillation followed by any of the tested NIV techniques was associated with a significant improvement of the arterial oxygenation. It is worth mentioning that the combined treatment of early surfactant followed by SNIPPV was found to maximize the initial response to surfactant therapy, achieving a rapid and uniform recovery of the baseline PaO_2_ values, a significant improvement of the VEI, a marked increase of the C_dyn_, and the best histological outcomes among all groups.

The use of NCPAP without surfactant treatment has become a standard clinical practice for the treatment of mild-to-moderate RDS [3,12,18]. Moreover, nasal ventilation has demonstrated great clinical value not only in preventing extubation failures [31,32] and as a treatment of apnea of prematurity [33], but also as a first line treatment against RDS [34,35]. Unfortunately, in many cases the application of NIV alone is not enough to adequately support RDS infants, particularly those born before the 28^th^ week of gestation, in which the NCPAP failure fraction stands at approximately 40–50% [18]. In the present study, neither NCPAP, NIPPV nor SNIPPV alone could revert the respiratory failure induced after BALs, which indicates a marked depletion of the surfactant pool with the applied lung lavage protocol [28]. Irrespective of the ventilation modality, arterial oxygenation remained low in all groups treated with NIV only. The histological scores were as well significantly worse in the NIV only treated groups compared the ones observed in their equivalent SF-treated groups. Of note, the mean PaCO_2_ of the NCPAP group, at the end of the follow-up period, was significantly higher compared to the groups ventilated with intermittent positive pressure. We attribute this difference to the higher capacity of NIPPV and SNIPPV to flush the CO_2_ from the airways.

The results from this study suggest that, in the setting of a more severe RDS, early surfactant followed by NIV, avoiding intratracheal intubation, might represent the most effective therapeutic approach. Several attempts have been made to deliver a dose of surfactant without intubation. Surfactant aerosol delivery was developed with the aim to administer surfactant in combination with NIV. The safety and the efficacy of this approach were investigated in small clinical studies [23–26]. Although the therapy was proven to be safe, the clinical efficacy of this technique is not yet convincing [36]. Surfactant administration using the laryngeal mask airway has also been investigated as a less invasive surfactant administration method [37]. Recently, a minimally invasive way of surfactant administration has been successfully implemented in preterm infants: this technique consists of delivering an intratracheal bolus of surfactant through a thin tube, while the infants are supported with NCPAP [19,20]. This approach reduces the need for mechanical ventilation, supplemental oxygen, and NCPAP. Most of the aforementioned less invasive surfactant administration techniques used NCPAP immediately after surfactant delivery. The use of NCPAP following surfactant instillation was also associated with a rapid and uniform improvement of oxygenation in our *in vivo* model. However, the results obtained in this study bring up the following scientific question: should SNIPPV rather than NCPAP be used as a primary non-invasive respiratory support immediately after surfactant administration? The available clinical evidence remains controversial. Chen *at al*. did not find any statistical differences in the rate of endotracheal ventilation in twin pairs receiving surfactant and supported with either NCPAP or NIPPV [38]. Nevertheless, they found a marginal statistically significant difference favouring the use of NIPPV in infants with a gestational age of 32–33 weeks. Of note, the study by Chen *et al*. did not use breath-synchronization. Gizzi *et al*. compared the chart data of 31 infants undergoing the InSurE (Intubate-Surfactant-Extubate) approach supported with NCPAP with the data of 33 infants undergoing InSurE but supported with SNIPPV [39]. In this single-centre study, the use of SNIPPV significantly reduced the InSurE failure (2/33 in the SNIPPV group vs. 11/31 in the NCPAP group, *P* < 0.004) and the need for mechanical ventilation. These clinical studies suggest that the use of nasal ventilation might be of advantage compared to NCPAP after SF administration. In particular breath-synchronization has the potential to maximize SF-therapy and the overall pulmonary ventilation. For instance, during non-synchronized NIPPV, effective patient-ventilator synchronization has been described to occur only in 25% of the cycles [40]. The remaining asynchronous cycles are delivered by the ventilator at all phases of the infant’s respiratory cycle, including the early-, mid- and late-expiration [41]. This mismatch alters the spontaneous respiratory rhythm, induces glottal narrowing, increases the work of breathing (WOB), and can ultimately cause volutrauma [41]. Conversely, during SNIPPV, the positive pressure is delivered by the ventilator with the opened glottis and is therefore effectively transmitted to the lungs, reducing the thoraco-abdominal asynchrony, the inspiratory effort, and the WOB, yielding an improved pulmonary ventilation [17].

In the present study, the most prominent pulmonary improvement among all SF-treated groups, as well as the lowest lung injury scores, were also observed when SNIPPV was applied right after surfactant instillation. Therefore, this study is in line with the clinical reports that found a benefit in synchronizing the ventilator’s drive with the patient’s spontaneous breathing efforts. To achieve an efficient synchronization, we used the Graseby abdominal pneumatic capsule; this device has shown to correctly detect inspiration 88% of the times [42]. Compared to non-synchronized NIPPV, the use of SNIPPV immediately after surfactant instillation was associated with significantly better PaO_2_, VEI, and Cdyn values. In addition, the SNIPPV+SF group had a significantly lower injury score compared to the NIPPV+SF group and showed the best outcomes for all the studied items (inflammation, hemorrhage, edema, atelectasis) among all the study groups. We speculate that the coordinated “breaths” delivered by the ventilator during SNIPPV might have helped spreading the surfactant immediately after instillation, accounting for a better initial pulmonary distribution of surfactant that led to a rapid increase of the PaO_2_, a significant improvement of the VEI, and a better histological assessment.

We acknowledge that the present *in vivo* study has several limitations: on the one hand, the surfactant-depleted adult rabbit does not completely resemble the complex pathogenesis of RDS, which takes places in a significantly larger time-scale compared to the relatively short follow-up of our protocol. On the other hand, PaCO_2_ values remained above physiological levels in all groups, irrespective of the applied treatment. We assume that the high PaCO_2_ levels are due to the increased resistance to air flow of the customized nasal cannulas, which have been originally designed for humans and not for rabbits. Nevertheless, we have implemented an *in vivo* model of surfactant deficiency that can be alternatively managed with NCPAP, NIPPV, or SNIPPV; this model resembles a moderate respiratory distress, which cannot be reverted by merely applying NIV support. Conversely, surfactant administration followed by NIV was associated with a rapid improvement of arterial oxygenation, making this model suitable for the preclinical testing of alternative surfactant delivery methods, e.g. surfactant nebulization and laryngeal mask surfactant administration [37,43]. The present study also suggests that SNIPPV applied immediately after surfactant instillation may maximize the effects of surfactant therapy compared to other NIV modalities.

## Supporting information

S1 Fig Examples of histological findings in surfactant-depleted rabbits managed with non-invasive ventilation. Histological examples of lung parenchyma presenting inflammatory infiltrate, hemorrhage, edema and atelectasis. Haematoxylin & Eosin staining.
(PDF)
