# Supplementary figures and images for: Surfactant replacement therapy in combination with different non-invasive ventilation techniques in spontaneously-breathing, surfactant-depleted adult rabbits
Source: PLoS One. 2018 Jul 12;13(7):e0200542. doi: 10.1371/journal.pone.0200542 (PMC6042776; doi:10.1371/journal.pone.0200542)

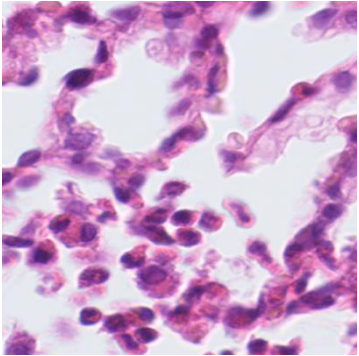

**Inflammation**

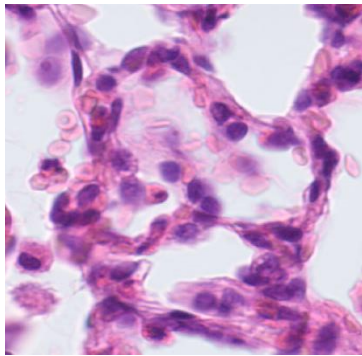

**Hemorrhage**

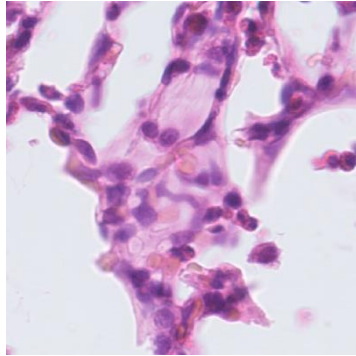

**Edema**

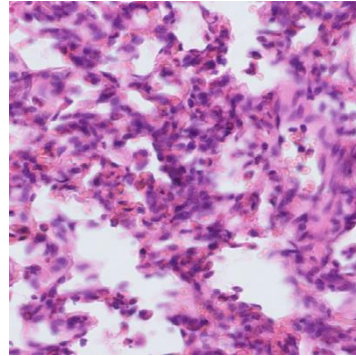

**Atelectasis**

**S1 Fig. Examples of histological findings**

Supplement: S1 Fig — Histological examples of lung parenchyma presenting inflammatory infiltrate, hemorrhage, edema and atelectasis. Haematoxylin & Eosin staining. (PDF) [file pone.0200542.s001.pdf]
